# Food Insecurity and Nutritional Inadequacy in Children and Adolescents of Basic Education Schools of Cantagalo District in São Tomé and Príncipe, Central Africa

**DOI:** 10.3390/nu16162802

**Published:** 2024-08-22

**Authors:** Francisca Ferreira, Maria Tavares, Renata Barros, Cláudia Camila Dias, Rita Morais, Madalena Ortigão, Patrícia Padrão, Mónica Rodrigues, Pedro Moreira

**Affiliations:** 1 Faculty of Nutrition and Food Sciences, University of Porto, 4150-180 Porto, Portugal; francisca.ferreira.3005@gmail.com (F.F.); renatabarros@fcna.up.pt (R.B.); ritasalomemorais@gmail.com (R.M.); patriciapadrao@fcna.up.pt (P.P.); 2 Helpo-Non-Governmental Development Organization, 2750-318 Cascais, Portugal; mariatavares.mmt@gmail.com (M.T.); madalenaortigao@helpo.pt (M.O.); 3 Epidemiology Research Unit, Laboratory for Integrative and Translational Research in Population Health, Institute of Public Health, University of Porto, 4050-600 Porto, Portugal; carolina101997@hotmail.com; 4 CINTESIS@RISE, Department of Community Medicine, Information and Health Decision Sciences (MEDCIDS), Faculty of Medicine, University of Porto (FMUP), 4200-450 Porto, Portugal; camila@med.up.pt; 5 Knowledge Management Unit, Faculty of Medicine, University of Porto (FMUP), 4200-319 Porto, Portugal

**Keywords:** food insecurity, nutritional inadequacy, children, adolescents, households, Central Africa

## Abstract

Food insecurity (FI) is a critical socioeconomic and public health problem globally, particularly affecting children’s nutritional status and development. This cross-sectional study aimed to assess the prevalence of nutritional inadequacy among children and adolescents in the Cantagalo district of São Tomé and Príncipe (STP), in Central Africa. It also assessed their households’ FI situation and examined sociodemographic, anthropometric, and nutritional characteristics associated with severe FI. Data included 546 children/adolescents (51.8% males, aged 9–15 years) from the eight public basic education schools. A structured questionnaire provided sociodemographic data, while anthropometric measurements assessed nutritional status. Dietary intake data were gathered using a single 24 h dietary recall, and the adjusted prevalences of nutritional inadequacy were obtained using version 2.0 of the PC-Software for Intake Distribution Estimation (PC-SIDE^®^). The Household Food Insecurity Access Scale was used to assess FI, and households were classified as severely or non-severely food insecure. Multivariable binary logistic regression models adjusted for potential confounders identified factors related to FI. Children’s/adolescents’ thinness was exhibited in 34.1% of participants, and over 95% had inadequate intake of essential micronutrients, including iron. Notably, 73.7% were severely food insecure. A higher severity of FI was positively associated with a lower intake of iron and certain household head characteristics, such as being female or older, and negatively associated with having a home garden.

## 1. Introduction

Safe and sufficient access to food is still today a distant reality for billions of households worldwide, who are denied food security (FS). FS is defined by the Food and Agriculture Organization of the United Nations (FAO) as the condition which occurs “when all people, at all times, have physical, social and economic access to safe and sufficient food to meet their dietary needs and food preferences for an active and healthy life” [1]. Its multifactorial etiology contributes to the complexity of FS, which considers four dimensions: availability, access, utilization, and stability [2]. When one or more dimensions are affected, a situation of food insecurity (FI) occurs.

Factors such as the age, sex, educational attainment, and marital status of the head of the household, as well as its size, family income, social support, children’s age, and level of dependency influence FI [3,4,5,6]. External factors, such as conflicts, climate extremes, seasonality, and economic instability, exacerbate FI situations [7,8], which worsened during the coronavirus disease 2019 (COVID-19) pandemic [8,9], with one-third of the world’s population being deprived of adequate nutrition [8]. São Tomé and Príncipe (STP) is a lower–middle-income country, whose progress has been constantly impeded by some of those factors, such as climatic variability (heavy rain, extreme heat, and storms), geographical (insularity and remoteness), socioeconomic (highly dependence on food imports, inflation, unemployment, and uncontrolled urbanization), and health obstacles (limited healthcare resources and a serious lack of proper hygiene and sanitation conditions) [10]. Worldwide, in 2023, one in five people faced an acute FI situation, twenty-four million more people than in the previous year [8], and vulnerable groups include children, women (2.4% more than men), and rural populations (33.3% versus 26.0% in urban areas in 2022), differently [11]. At the current sluggish rate of change, the numbers of FI are expected to remain regionally high and, by 2030, chronic malnutrition will affect approximately 600 million people [7].

Africa faces higher prevalences of FI than other parts of the world [12], with 61% of households experiencing moderate to severe FI and 19.7% of Africans being undernourished (versus 9.2% in the world) [12]. Africa is also a continent in nutritional transition [13], with changes in dietary patterns, particularly worrying in childhood and adolescence. In these poorer countries, we are witnessing the phenomenon of the “triple burden of malnutrition” [14], in which the extremes of undernutrition and overweight/obesity are emphasized, often in conjunction with hidden hunger [15]. Hidden hunger is characterized by micronutrient deficiencies, especially vitamin A, iron, iodine, zinc, and folate [14]. Globally, one in two preschool children and two in three women of reproductive age are deficient in at least one of the nutrients mentioned [16]. These figures are even more alarming in South Asia and sub-Saharan Africa, of which STP is a part [13]. The permanent and damaging impact of hidden hunger is most critical during periods of growth and development [17], contributing to poorer health outcomes, worse school performance, and limited future opportunities, which perpetuate poverty [17,18]. Poverty limits the availability and access to healthy foods, often leading people to purchase cheaper, energy-dense, and nutrient-poor alternatives [19]. This contributes to poor child development outcomes, including anemia, obesity, and mental health issues [18,20,21].

In STP, the FI situation has been deteriorating every year. In 2021, the prevalence of moderate and severe FI reached 54.6%, of which 14.1% corresponded to the highest level of severity [22]. In 2023, 7581 households lived in extreme poverty, 73% of which were female-headed [23], and these alarming statistics highlight the urgency for targeted research like the present study.

It is, therefore, essential to have current data on the nutritional status and FI in Santomean children and adolescents. Thus, this study aims to assess the prevalence of nutritional inadequacy in Santomean children and adolescents, from the Cantagalo district, as well as their household’s FI status, seeking to identify possible anthropometric, sociodemographic, and nutritional characteristics associated with higher severity of FI.

## 2. Materials and Methods

### 2.1. Study Design and Participant Assessment

This is a cross-sectional study using data from the “MeNutRic Mais: Improving the Nutritional Status of Children in São Tomé and Príncipe” project, conducted in Cantagalo District in STP, a low–middle-income country in Central Africa. This district was selected for this study following the ongoing work of the Helpo Non-Governmental Development Organization, which has been implementing health, education, and nutrition programs in several communities of STP for several years, with a focus on vulnerable populations. The Helpo Non-Governmental Development Organization project aims to improve the quality of school meals through food education tools, school gardens, and pig farming. The data were collected by a multidisciplinary team with trained interviewers between November 2022 and February 2023.

**Figure 1:**
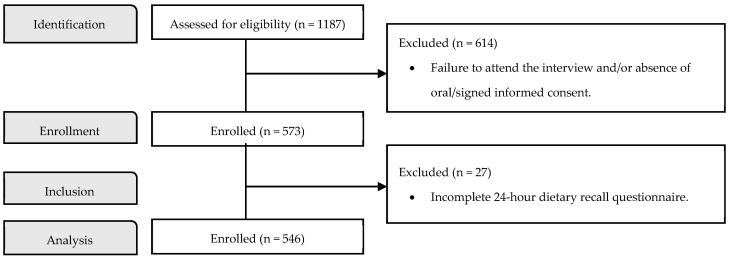
Study flow diagram.

The inclusion criterion was to be a 5th or 6th grader at one of the 8 public basic education schools covered by the project. Exclusion criteria were non-attendance at the interview and/or the absence of data essential to the research, particularly on dietary intake. The sample size was calculated by the National Statistics Institute (INE) of STP, using a cluster and convenience sampling method, thus guaranteeing the estimated numbers in each school in Cantagalo. Further analysis confirmed that there were no differences regarding food insecurity (FI) when comparing excluded participants to those included with complete dietary data, suggesting that our findings were not biased by the exclusion criteria. Accordingly, out of the total of 1187 eligible students, 573 attended the interview and were enrolled in this study. In the end, only 546 were analyzed, as they met all the criteria. Figure 1 presents the study flow.

This study was approved by the Health Ethics Committee for Scientific Research from the Ministry of Health of the Democratic Republic of São Tomé and Príncipe. Written or oral (in cases of illiteracy) informed consent was obtained from the parent/guardian who agreed to take part in the study. For participants who provided oral consent, this process was carefully structured to ensure comprehension and voluntariness.

### 2.2. Sociodemographic and Lifestyle Data

For the sociodemographic and lifestyle characterization of the families, questionnaires were applied to the guardian addressing the following information: relationship of the guardian to the child/adolescent, sex, age, marital status, educational attainment, employment status in the last 12 months (classifying this variable in four categories, namely student, housewife/househusband, employed/self-employed, and unemployed/retired) and smoking habits of the head of household, the number and ages of household members living together, monthly family income in Santomean Dobras (STN- official national currency), and the average daily amount spent on food. It was also asked who was responsible for cooking meals and whether there was a garden at home.

For the sociodemographic and personal history characterization of the child/adolescent, information was obtained on their sex, age, year of schooling, number of siblings and birth order, mother’s age at birth, occurrence of breastfeeding (whether or not they were breastfed), and age of weaning (in months). Question content also covered the main source of the food consumed, whether they ate breakfast on a daily basis, took snacks to school, ate the school meals and how often they ate those on a weekly basis, how often they bought other foods in and/or around the school (up to 100 m from the school gate) on a weekly basis, and how much they spent. Information was also obtained on the consumption, origin, and type of treatment of the water usually drunk during the school day, as well as the mode and duration of home–school and school–home journeys during a typical week. Finally, questions were asked about sleeping habits and physical activity and/or sports and active play.

### 2.3. Anthropometric Assessment

The anthropometric assessment of the children/adolescents was carried out by trained nutritionists, following standardized procedures [24]. To measure weight (in kilograms, kg), a calibrated Seca^®^ model 877 digital scale (0.1 kg resolution) was used, with the child/adolescent wearing no shoes and minimal clothing. Height (in meters, m) was measured using a Seca^®^ model 213 portable stadiometer (1 mm resolution), with the participant barefoot, in an anatomical position and their head positioned in the Frankfort Plane. Considering its ease of use and accessibility, and also minimizing error in data entry and analysis, we used World Health Organization (WHO) AnthroPlus^®^ software (for school-aged children and adolescents from 5 to 19 years) version 1.0.4, to calculate height-for-age z-score (HAZ) and Body Mass Index-for-age z-score (BMI-for-age z-score), in standard deviation (SD), and classify these data according to the 2007 WHO growth references [25]. Using version 1.0.4 WHO AnthroPlus^®^ software, height-for-age z-score (HAZ) and Body Mass Index-for-age z-score (BMI-for-age z-score), in standard deviation (SD), were calculated and classified according to WHO growth references [25]. By checking the Personal Child Health Record booklet, birth weight was characterized according to the 2006 WHO Child Growth Standards [26].

### 2.4. Dietary Intake Assessment

Dietary intake was gathered by trained nutritionists using a single 24 h dietary recall (24HDR). A detailed description of all the food and drink ingested was recorded, specifying the time and place of meals, quantities, brands of products, and the use of seasonings/spices and broths in composed dishes. In order to assist in the process of quantifying food, we used household measures, food models and photographs.

The dietary data were converted into nutritional information using the Food Processor^®^ nutritional analysis software version 11.11.32. Nutritional information on local products and recipes was added to the software in order to consider the typical Santomean gastronomy. The 2019 Food Composition Table for Western Africa (WAFCT) [27] and the Portuguese Food Composition Table 6.0 (TCA) [28] were considered, as well as the direct reading of food labels. During data entry, the information was carefully analyzed, corrected, and revised to minimize errors in estimating nutritional distribution. Thus, considering the available nutrients, total energy, total fat, saturated fat, monounsaturated and polyunsaturated fat, carbohydrates, total sugars, total and soluble fiber, protein, cholesterol, beta-carotene, vitamins A, B-1, B-2, PP, B-6, B-12, C, D, E (α-tocopherol), K, folate, and iron, magnesium, calcium, zinc, copper, phosphorus, potassium, selenium, and sodium were analyzed.

In order to estimate the prevalence of inadequate nutritional intake in the population, the harmonized nutrient reference values for populations (H-NRVs) were considered, particularly the harmonized average requirements (H-ARs) and the harmonized upper level (H-UL) [29]. The H-ARs are the result of adapting either the European Food Safety Authority (EFSA) average requirements (ARs), or the Institute of Medicine/National Academies of Sciences, Engineering, and Medicine (IOM/NASEM) estimated average requirements (EARs). Whenever the AR or the EAR are undefined for a certain nutrient, then the 0.8*AI (adequate intake) calculation is used, prioritizing the most recent value [29]. For H-UL, the EFSA values are used, given their more conservative and toxicity-preventive nature [29].

The crude prevalences of nutritional inadequacy were obtained using the “EAR cut-off point” method [30], in this study, comparing the children’s/adolescent’s intake with the corresponding H-AR, and are available in Appendix A (Table A1). In order to estimate the adjusted prevalence of nutritional inadequacy, we used the version 2.0 of the PC-SIDE^®^ (PC-Software for Intake Distribution Estimation) [31] software, developed by researchers from the Department of Statistics of Iowa State University (ISU). It allows us to estimate the distribution of usual nutrient intake and the prevalence of inadequate intake in a population [31]. It relies on complex statistical methods and, by adjusting the usual dietary intake distribution for demographic factors (such as sex and age) and intra-individual variability [32], PC-SIDE^®^ permits the implementation of the “EAR cut-off point” method (in this version, we used the H-ARs as the cut-points) to estimate the adjusted prevalence of inadequate intake. The percentage of intake below the H-AR was used as an indicator of the risk of inadequate intake. Children and adolescents were first divided into 2 groups according to their ages (group 1, aged 9–10, and group 2, aged 11–15), for which the prevalence of inadequacy was expressed. For each group, the H-NRV of the closest age category was assumed. As only a single 24HDR questionnaire was collected, we use external within-person variance estimates (unpublished and calculated by researchers at ISU [32]) obtained from the combination of three waves of the National Health and Nutrition Examination Survey (NHANES 2003–2004, 2005–2006, 2007–2008), so that the data could be analyzed. Consequently, the adjusted prevalence of inadequacy was obtained for protein, vitamins A, B-1, B-2, PP, B-6, B-12, C, D, E and folate, iron, magnesium, calcium, copper, selenium, zinc, and phosphorus (nutrients with a defined H-AR considered in the analysis of nutritional inadequacy).

The Goldberg method [33] was used to detect the presence of misreports in the 24HDR questionnaires, which identified 111 under-reporters, 419 plausible reporters, and 9 over-reporters. However, given the current context of poverty and hunger in STP and the lack of significant differences in the distribution according to FI status, all the children and adolescents were included in the nutritional analysis and the logistic regression was adjusted for this variable.

### 2.5. Household Food Insecurity Access Scale (HFIAS)

In order to subjectively assess the FI situation of households, the HFIAS was applied indirectly to parents/guardians. This tool refers only to the Access dimension of FI, and has been translated, adapted, and validated for multiple international contexts [34], including low- and middle-income countries such as STP [35,36]. It is based on the premise that the experience of FI causes predictable behavioral changes and reactions in families, which can be measured and quantified using a scale [34]. Furthermore, it also permits the characterization of the self-perception of the FI experience in a quick, practical, and economic manner [34,35]. It consists of 9 questions of occurrence (QO: yes or no) and 9 questions of frequency-of-occurrence [QFO: rarely (1 or 2 times), sometimes (3 to 10 times) or often (more than 10 times)], asked as a follow up to a positive QO. The severity associated with each QO is increasing. Age is not discriminated and the recall period is 30 days. Among the various indicators [34], the following were analyzed:

- Domains. Anxiety and uncertainty (with respect to food supply); insufficient quality (including variety and food preferences); insufficient food intake and inherent physical consequences [37].
- Score (of each household and average of the sample scores). It is scored from 0 to 27 points, according to the sum of the answers to the QFO. A score of 0 corresponds to a household that answered “No” to all the QOs (and consequently did not answer the QFOs), while a score of 27 reflects a total of 9 “often” answers (3 points) to the QFOs. As a continuous measure, the score is a more sensitive indicator of slight changes in the FI condition and its value is directly proportional to the level of insecurity.
- Prevalence. Prevalence of households in each of the 4 levels of food security (food secure, low, moderate, and severe FI).

Note that, hereinafter, whenever household FI is stated, it is exclusively referring to the Access dimension.

### 2.6. Statistical Analysis

For categorical variables, descriptive statistics consisted of calculating absolute (*n*) and relative (%) frequencies. For continuous variables with a normal distribution, the mean (M) and standard deviation (SD) were calculated, while, for discrete and/or continuous variables with a non-normal distribution, the median (Mdn) and the 25th and 75th percentiles (P25–P75) were determined. Histograms were used to visually determine the normality of the distributions of continuous variables.

The sample was then stratified dichotomously into “no severe FI” and “severe FI”, according to the household’s FI status. To analyze the differences between the groups, the t-student test for independent samples (if continuous variable with a normal distribution), and the Mann–Whitney U test (if discrete variable and/or with a non-normal distribution) were used. Pearson’s chi-squared test (*χ*^2^) or Fisher’s test (in cases where the *χ*^2^ test’s assumptions were not met) were used for categorical variables.

Multivariable binary logistic regression models were applied to assess possible associations between the highest severity of FI (dependent variable) and the sociodemographic (relationship of the parent/guardian to the child/adolescent; sex, age, relationship to the child/adolescent, educational attainment and employment status of the head of the household, household income and amount spent on food, number of household members living together, and having a home garden), anthropometric (birth weight), individual (drinking water while at school) and nutritional variables considered relevant, as well as adjusting for other covariates suggested by the literature (child/adolescent’s sex, age, and BMI-for-age z-score). A new dichotomic variable “being a misreporter” was considered as a possible candidate variable, but was excluded in the final model. For the analysis, certain categories of some variables were combined and the Hosmer–Lemeshow test was performed to evaluate the goodness of fit of the model. By using the backward stepwise method and selecting the best Receiver operating characteristic (ROC) curve, the most discriminative and best fitting model was obtained.

Statistical significance was assumed for a *p*-value of <0.05 and a 95% confidence interval (CI). All the statistical analyses were performed using the International Business Machines (IMB) Statistical Package for the Social Sciences (SPSS^®^) Statistics software version 28.0.1.0 for Windows^®^.

## 3. Results

Table 1 presents the families’ sociodemographic characteristics according to FI status. The mother was the guardian for most of the children/adolescents. Regarding the household, 87.5% were female-headed, with the mother being the head in 72.9% of them. The mean (±SD) age of the head of the household was 38.1 (±9.8) years, the majority cohabited with a partner (73%), and less than 1% were smokers (0.4%). Almost 40% of the heads of the household only had the 1st cycle of basic education (1st to 4th grade), 2.5% had no education, and only a minority (1.1%) had completed higher education. At the time and at 12 months prior to the interview, the majority were employed (48.8%), 34.4% were housewives/househusbands, 14.4% were unemployed or retired, and only 2.4% were still studying. Six (five–seven) was the median (P25–P75) number of household members living together. Approximately 32% of the households had a monthly income of between 2000 and 3000 STN, and 7.7% had less than 500 STN per month. Moreover, more than half of the households (55.3%) reported a daily spending of between 100 and 200 STN on food.

In most households (85.3%), one of the parents was the member in charge of food preparation and cooking. Lastly, over 86% of the households did not have a home garden.

When comparing families’ characteristics according to the household’s food insecurity status (Table 1), statistically significant differences were found for the following variables: the relationship of the guardian to the child/adolescent; the head of the household’s sex, age, education, and employment status; the household income; and the amount spent on daily food expenses, as well as having a home garden or not.

As reported in Table 1, severe food insecurity was more prevalent in households with mothers as guardians (78.8% vs. 59.1%), and as the head of the household (76.9% vs. 60.2%), and, consequently, with a female as the head of the household (90.8% vs. 79.7%). Additionally, severely food insecure households were headed by older members (38.91 ± 10.01 vs. 35.77 ± 8.97), non-educated (3.4% vs. 0%) or with a lower level of educational attainment (up to 6th grade: 66.2% vs. 43.5%). The highest level of severity of FI was more prevalent when the head of the household was either a housewife/househusband (34.2% vs. 25.8%) or unemployed/retired (17.6% vs. 10.9%) and in households with monthly incomes of less than 500 STN (10.3% vs. 2%) that did not have a home garden (88.5% vs. 78.7%).

Regarding children and adolescents (Table 2), approximately 52% were male, with a mean age of 10.8 (±1.2) years, aged between 9 and 15 years, and 56.2% of them were in the 5th grade. The median (P25–P75) number of siblings was three (two–four), and the majority were first or second born children (39.7% and 24.1%, respectively). At birth, their mothers’ median (P25–P75) age was 25 (21–31) years. Overall, approximately 87% were identified as term babies, and the mean newborn weight was 3219 (±619) grams. The majority (83.7%) were born with a normal weight for gestational age, while less than 9% were born with a low or very low birth weight. Nearly all (98.5%) were breastfed, with a median (P25–P75) breastfeeding duration of 18 (15–20) months. At the time of the interview, the mean (±SD) HAZ and BMI-for-age z-score were −0.50 (±1.08) and −0.66 (±0.98), respectively. Almost 92% had a normal height for their age, but 7.1% were considered stunted. At the time of the data collection, the majority of the children/adolescents had a normal weight (61.4%). However, a considerable prevalence of thinness was identified, including mild (27.3%), moderate (6%), and severe (1%) thinness. The prevalence of overweight and obesity was less than 5%.

In the 12 months prior to the study, approximately 55% of the children/adolescents took at least one form of dietary supplement. Even though food purchases (90.5%) were the main source of nutrition, 6.4% still depended on family production, farming, hunting or fishing, and around 3% depended on food exchanges (including for work), donations from family/friends, or food assistance.

With regard to their eating habits, the majority (91.7%) reported eating breakfast at home on a daily basis. About 93% ate a hot meal for lunch at school, but only 62.3% did on a daily basis.

Almost half of the children/adolescents did not bring snacks to school (from home) and, as such, approximately 88% bought foods/snacks, mostly in the surrounding areas outside school (54.6%). Median (P25–P75) spending was 5 (2–6) STN and once or twice a week was the most reported frequency of these purchases (35.6%), with some children/adolescents (2.7%) buying more than once a day. Approximately 90% reported drinking water while at school, mainly from fountains or wells (45%) or bottled water (34.8%). Of the 263 children/adolescents who knew how to recognize some type of water treatment, more than 45% reported not having access to treated water. On a typical school week, almost every child/adolescent walked to school and returned home in the same way. The median (P25–P75) duration of both commutes was 20 (10–40) minutes. In relation to lifestyle habits and sleep patterns, the median (P25–P75) daily time spent on physical activity and/or sports and/or active playing was 120 (60–180) minutes, and the median sleep duration was (P25–P75) 11 (10–11) hours.

The previous characteristics display no statistically significant differences according to food insecurity status, except for birth weight and drinking water while at school. In severely food insecure households, children/adolescents were born with a lower birth weight than in non-severely food insecure ones (3172 ± 570 vs. 3308 ± 694 g). Additionally, during school time, there were more non-water drinkers from households with the highest level of severity (11.7% vs. 4.7%).

The nutritional intake distributions of children and adolescents showed no statistically significant differences between households with no severe food insecurity and severe food insecurity (Table 3).

Table 4 summarizes the adjusted prevalences of nutritional inadequacy among children and adolescents. With the exception of protein, niacin, and vitamin B12 (all ages), vitamins C and E, magnesium, phosphorus, and selenium (in 9–10-year-old participants), the prevalences of inadequacy were very high, with most exceeding 70%. Notably, for vitamins A, B2, folate, iron, calcium, zinc, copper (all ages), vitamin B6, and phosphorus (in 11–15 years old participants), more than 95% had inadequate intakes. For the nutrients considered in the analysis, none of the children/adolescents had intakes above the H-ULs, and as such, these are not presented.

The HFIAS questionnaire indicators are presented in Table 5. With regard to the HFIAS domains, the prevalence for “anxiety and uncertainty” stood at 76.5%, “insufficient quality” at 93%, and “insufficient food intake and respective physical consequences” at 90.3%. The mean (±SD) HFIAS score was 13.57 (±6.67). The findings demonstrated that, during the 30-day recall period, only 5.1% of the households experienced food security. Amongst food-insecure households, the largest proportion reported experiencing severe food insecurity (73.7%), with the remaining reporting moderate (19.5%) and mild (1.6%) FI.

Statistically significant differences between non-severely and severely food-insecure households were found for the three domains mentioned above, as well as for the HFIAS score. Severely food insecure households have experienced feelings of anxiety and uncertainty about their food supply almost twice as much (84.1% vs. 48.4%) and have adopted more behaviors, which reflect insufficient food quality (97.8% vs. 77.3%) and insufficient food intake (99.7% vs. 61.7%). As expected, severely food insecure households had a significantly higher HFIAS score (16.18 ± 5.27 vs. 6.40 ± 4.52).

In the multivariable binary logistic regression model, the associations between severe food insecurity in participants’ households and the different sociodemographic, lifestyle, and nutritional characteristics, adjusted for several confounders, are detailed in Table 6, as these are particularly important factors, such as being a female or an older head of the household, having no home garden, and presenting a lower iron intake.

According to the analysis, female and older–headed households (OR: 5.505; 95%CI 2.209–13.721 and OR: 1.045; 95%CI 1.008–1.084, respectively), and children/adolescents with higher intake of magnesium (OR: 1.013; 95%CI 1.004–1.021) had higher odds of being severely food insecure. On the other hand, the results revealed that having a garden at home significantly lowers the odds of being severely food insecure (OR: 0.343; 95%CI 0.157–0.750). In addition, children’s/adolescents’ lower intakes of iron and carbohydrates were significantly associated with being severely food insecure (OR: 0.735; 95%CI 1.004–1.021 and OR: 0.995; 95%CI 0.991–1.000, respectively).

## 4. Discussion

To the best of our knowledge, this is the first study to highlight the adjusted prevalence of nutritional inadequacy among children and adolescents in STP, and the association between higher severity of FI and nutritional intake.

The majority of children/adolescents demonstrated having inadequate nutritional intakes for half the 18 nutrients analyzed, including vitamin A, folate, iron, and zinc, which are crucial micronutrients for optimal health, growth, and development [38,39], and whose deficiencies typically characterize hidden hunger [17]. The nutritional inadequacy data for pediatric age are very scarce, worldwide, due to the persistent absence of these age groups across research, especially in low- and middle-income countries. Nonetheless, some studies conducted in those countries indicated a wide range of micronutrient deficiency prevalences, ranging from 0% to 55.1% for Vitamin A in Nigeria (from sub-Saharan Africa), and from 3 to 75.6% for zinc in Kenya and South Africa [40]. The latest data suggest that anemia affects 64% of pre-school children from sub-Saharan Africa, with iron deficiency being the cause in the majority of the cases [41].

Our study revealed a significant association between higher severity FI and a lower iron intake, even after adjusting for several confounders (OR: 0.735; 95%CI 1.004–1.021). This finding highlights a critical public health issue, considering iron’s crucial role supporting growth and cognitive development and in preventing anemia from occurring; this is a widespread public health problem affecting mainly children and women of reproductive age, especially in rural and poor contexts, in Africa [42,43]. During adolescence, a higher consumption of ultra-processed, energy-dense and nutrient-poor foods, along with more food choice autonomy, may also enhance iron deficiency [44,45].

Although the multivariable binary logistic regression model also indicated statistically significant associations between higher severity FI and children’s/adolescents’ intake of carbohydrates and magnesium, the results are so marginal that it is difficult to attribute any practical relevance.

The head of the household´s sex was clearly shown to be a correlated factor of FI severity, with female-headed households having 5.5 more odds of experiencing severe FI than male-headed ones. Although it cannot be directly compared, due to the use of different FI assessment tools, similar results have been found in other studies [46,47], which took place in low- and middle-income countries historically recognized as patriarchal societies [46]. In 2021, 67% of Santomean households were female-headed, with poverty levels close to 70% [10]. Our study showed an even higher prevalence of 88%, with only a 48.8% employment rate among these households. Almost all (91%) of the severe FI households were headed by females. These findings are in line with the literature, which addresses the feminization of poverty [48], markedly in low- and middle-income countries [12]. Currently, despite the growing influence of women in the labor market, sociocultural and sex-based job inequalities are still notable, compromising income and FS status and making female-head households more vulnerable to FI [49].

Our study also suggests that households headed by older people had slightly higher odds of being severely food insecure (OR: 1.045; 95%CI 1.008–1.084). Some studies conducted in low- and middle-income countries are in line with our findings, highlighting the fewer job opportunities available (due to retirement and disability), precarious salaries, and comorbidities as possible vulnerabilities leading to FI [50,51]. Contrasting results were found in studies conducted in Kenya and Nigeria, with the age of the head of the household being identified as a protective factor against FI, as more age usually represents more farming experience, which can be an advantage for the families’ FS status [52]. In STP, life expectancy at birth remains low (total: 69.2 years in 2022), and chronic Noncommunicable diseases (NCDs) represent 60% of the causes of mortality [10,53]. As such, older heads of households are likely to have more comorbidities and higher health costs, limiting the family income available for healthy, sufficient, and safe food.

Our study showed that children/adolescents from households with a home garden had significantly lower odds of being severely FI (OR: 0.343; 95%CI 0.157–0.750), aligning with the literature [54,55], which emphasized home gardens as crucial weapons against household hunger and FI. Home gardens may provide accessible, affordable, and nutritious foods [55], especially in rural areas, which tend to be further away from food retailers or markets; having a garden or land can therefore be a valuable strategy for increasing household FS [56]. In critical periods of economic and climatic difficulties, having a home garden can be a coping strategy to minimize a family’s feeding difficulties [56,57] and provide social and economic benefits, by strengthening family relationships and improving resource management, as well as self-sufficiency [58]. Despite the positive impact of home gardens, our data indicated that only 11% of the households involved had a home garden. Notably, nearly 89% of the households experiencing severe FI did not have one. School gardens have proven to be a valuable food educational tool, improving students’ eating habits, self-esteem, social skills (teamwork), and levels of physical activity by providing active outdoor experiences [59,60]. In STP, projects such as “MeNutRic” in Lobata, in 2021, and “MeNutRic Mais” in Cantagalo, in 2022, which have been successful, suggest a need for identical strategies, or the expansion of these strategies, to optimize school meals’ nutritional quality.

Using the HFIAS, our findings identified alarmingly high prevalences of FI. The majority (94.9%) of the households were affected with some degree of FI, with approximately 74% being severely food insecure. These prevalences are slightly higher than those reported in the latest national data (moderate and severe FI ≈ 55%, 2021) [61], even though different measurements have been used to assess FI. In our study, the HFIAS score was significantly higher in severely food insecure households. As the most sensitive indicator of FI, directly related to the degree of severity, this score strengthens the importance of using practical scales such as the HFIAS for screening, diagnosis, and monitoring FI situations [34], in STP. Future research, using nutritional biomarkers and other health measures, should try to confirm these findings, particularly in relation to iron deficiency.

The intention was also to explore other socioeconomic, lifestyle, anthropometric, and health characteristics of children/adolescents and their families associated with their FI status.

With regard to education, heads of households experiencing severe FI had lower levels of educational attainment (non-educated or up to 6th grade), and the non-educated ones were all severely food insecure. In STP, pre-school and basic education are free, but only compulsory up to the 4th grade, which results in higher absenteeism and lower completion rates in the subsequent levels [62,63]. This reality is consistent with our findings, showing many adolescents between 11 and 15, beyond the expected age for 5th and 6th graders [63], indicating grade retention. Although no significant association was found between the level of education and FI, the role of education on household FS is critical, as higher levels of education are generally associated with better job opportunities and salaries, factors known to mitigate FI.

The employment status of the head of the household is recognized as an important predictor of FI [64,65]. Our study found that, in severely food-insecure households, there were significantly more housewives/househusbands or unemployed/retired participants. Nevertheless, no association was found between employment status and FI situations, which can be due to the fact of employment status misreport, since “employed” did not differentiate between employee, self-employed, and other forms of precarious or non-paid employment (such as work in exchange for food).

With regard to children’s/adolescents’ nutritional status, the prevalences of thinness and overweight/obesity were around 34% and 4%, respectively, differing from the latest available national statistics (2019), which indicated considerably lower prevalences of thinness (9%) and higher prevalences of overweight/obesity (8%) [66]. In STP, despite the decreasing trend in thinness prevalence in pediatric age, in 2019, the highest prevalence of overweight/obesity was recorded since 1990 [66], which may reflect the globalization and nutritional transition phenomena. Nevertheless, these data were obtained before the COVID-19 global pandemic and the Ukraine War, and further data are either scarce or non-existent for most low- and middle-income countries [8]. Evidence suggests that these humanitarian crises have contributed to the rise in the triple burden of malnutrition, especially in low- and middle-income countries and mainly in Africa [8,13]. Thus, the rise in global food prices during/after these periods limited the affordability and access to nutritious diets, worsening poverty and FI, and negatively impacting health and nutritional status [67]. Our findings might be among the first post-COVID/Ukraine War to reflect some of their detrimental effects on children’s/adolescents’ nutritional status. Moreover, children and adults from food-insecure households may have poorer eating and lifestyle habits, impacting their optimal nutritional status [20,21]. However, our study found no significant association between children/adolescents BMI-for-ages z-score and FI status.

Children/adolescents experiencing severe FI at home also reported significantly poorer water drinking habits at school, highlighting socioeconomic disparities in behavior regarding proper hydration [68]. Nearly half of the children (45.6%) drank untreated water, which reflects the poor access to safe water and sanitation in STP, despite abundant hydric resources [10], raising significant public health concerns.

For the majority of the considered nutrients, such as vitamin A, riboflavin, B-6, folate, iron, calcium, zinc, copper, and phosphorus, the children’s/adolescents’ adjusted prevalences of the inadequacy were alarmingly high. Despite National School Feeding and Health Program (PNASE)’s interventions in public schools providing food baskets, which led to improved students’ school performance, reduced school dropouts, and disability-adjusted life years (DALYs) of the benefiting students [63], in our study, only 60% of the children/adolescents ate school meals daily, even though there were no statistically significant differences according to the FI status. This low school meal acceptance may be attributed to peer influence and food preferences’ changes during adolescence, as well as other factors, such as food retail availability and advertising [69].

In the highlight of the findings, it is urgent to combat the transgenerational poverty, hunger, and FI that historically affects STP, by investing in younger generations. As a simple, practical, and cost-effective tool to assess FI, the HFIAS and its score indicator allowed us to obtain results which may be impactful for the monitoring of future health conditions in STP. Collaboration among healthcare providers, governments, and policy makers is required to provide universal and equitable access to a healthy, safe, and sustainable diet, to optimize the nutritional status of the whole Santomean population, to enhance local home and school gardens, to target vulnerable households with support programs (e.g., food aid, job initiatives), particularly those with lower-income, older, or female-headed households, to reduce gender inequality, and to assess the need for nutritional supplementation in vulnerable families.

Strategic plans to assess iron fortification practices, the expansion of iron supplements, and nutrition education strategies could significantly mitigate iron deficiencies. Thus, expanding the project of school gardens across all the schools in STP and encouraging their replication at home may promote healthier eating habits and food literacy, enhancing access to affordable and sustainable nutrient-rich diets. Additionally, limiting the availability of energy dense and nutrient-poor foods in and around schools and supporting local producers can further improve dietary choices and the overall eating environment.

This study has some limitations. Firstly, as a cross-sectional study, it was not possible to derive any causal relationship between a higher severity of FI and the variables examined. Secondly, a single 24HDR questionnaire was collected, which, although it is the less expensive and burdensome methodology to use in large surveys, may not accurately represent a typical day nor consider seasonal variations [70]. Moreover, children’s/adolescents’ concentration span and limited food knowledge make this methodology more challenging and may increase the likelihood of errors [71]. Ideally, multiple recalls would provide a more comprehensive representation of habitual intake. Anticipating this, the recall was indirectly applied by trained interviewers and our approach included detailed queries about portion sizes and the use of some food models to increase portion size accuracy and minimize misreporting errors [72], and the composition of mixed dishes, local food items, and specific commercial brands were also considered. Additionally, the use of the 24HDR did not interfere with the natural dietary behaviors of the participants, avoiding any alterations in their eating habits that might arise from the intrusive nature of more time-intensive dietary assessments [73]. Despite these challenges, a 24HDR remains a practical choice for assessing the typical diet of large groups [74]. Recognizing its limitations, BMI was used to characterize children’s/adolescents’ nutritional status, through height and weight collection. Nonetheless, objective measures of height and weight were obtained, avoiding parental self-perceptions, as parents tend to misjudge their children’s overweight/obesity category [75]. While our data collection was comprehensive, encompassing dietary intake, sociodemographic factors, and anthropometric characteristics, we did not specifically include or analyze chronic diseases beyond these parameters. We acknowledge that chronic diseases could play a significant role in influencing the relationship between food intake and health outcomes. Future research could be designed to investigate the impact of chronic diseases on these associations, providing a broader understanding of the interactions they could play. Additionally, the HFIAS was not specifically validated for the Santomean population, and, as an experience-based scale, its responses are very dependent on the respondents’ self-perception of FI, which may not be representative of the other members’ perceptions. Finally, even though our sample may be representative of all of 5th and 6th graders of Cantagalo District, it may not reflect the characteristics of the entire population of children and adolescents in STP.

This study also has strengths which increase our confidence in our findings. To our best knowledge, this is the first study to obtain adjusted prevalences of nutritional inadequacy among children and adolescents in STP, and to explore the associations between higher severity of FI and other sociodemographic, lifestyle, and nutritional characteristics. Also, our findings might be among the first post-COVID/War to reflect some of their detrimental effects on children’s/adolescents’ nutritional status. Thus, the Goldberg method was considered in the analysis, and, using PC-SIDE^®^, it was possible to estimate the usual nutrient intake distribution and adjust it for intra-individual variability. Moreover, our research included a large number of children and adolescents, ensuring the representativeness of the sample in Cantagalo, and the nutritional assessments were carried out by qualified professionals after local training and the information available in Food Processor^®^ was updated to include local products and recipes, minimizing the introduction of biases. Future research should track changes in FI and nutritional status over time, ultimately using more effective strategies to assess these complex dimensions and assess the impact of specific interventions aimed at decreasing FI and improving health status in STP.

## 5. Conclusions

Our findings indicated overall high prevalences of nutritional inadequacy with regard to essential micronutrients, with most exceeding 70% in both children and adolescents. This prevalence was higher than 95% for vitamin A, riboflavin, folate, iron, calcium, zinc, and copper in both children and adolescents, and for vitamin B-6 and phosphorus in adolescents. The prevalence of overall thinness was approximately 34% and the combined prevalence of overweight and obesity was less than 5%.

It is noteworthy that a higher severity of FI is positively associated with a lower intake of iron and some characteristics of the head of the household, such as being female or older, and is negatively associated with having a home garden.

## Figures and Tables

**Table 1:** Families’ sociodemographic characteristics according to food insecurity status.

	Total	Non-Severe FI	Severe FI	*p*-Value
Relationship of guardian (respondent) to the child/adolescent, *n* (%)				
Mother	390 (71.6)	75 (59.1)	272 (75.8)	<0.001
Father	44 (8.1)	15 (11.8)	22 (6.1)	
Grandmother/grandfather	28 (5.1)	4 (3.1)	22 (6.1)	
Uncle/aunt	41 (7.5)	13 (10.2)	24 (6.7)	
Other	42 (7.7)	20 (15.7)	19 (5.3)	
Head of the household				
Sex, *n* (%)				
Male	68 (12.5)	26 (20.3)	33 (9.2)	<0.001
Female	478 (87.5)	102 (79.7)	326 (90.8)	
Relationship to the child/adolescent, *n* (%)				
Mother	398 (72.9)	77 (60.2)	276 (76.9)	<0.001
Father	50 (9.2)	19 (14.8)	24 (6.7)	
Grandmother/grandfather	29 (5.3)	5 (3.9)	22 (6.1)	
Uncle/aunt	35 (6.4)	12 (9.4)	20 (5.6)	
Other	34 (6.2)	15 (11.7)	17 (4.7)	
Age in years, Mean ± SD	38.06 ± 9.79	35.77 ± 8.97	38.91 ± 10.01	0.002
Child caregiver, *n* (%)				
Yes	497 (91.0)	113 (88.3)	326 (90.8)	0.410
No	49 (9.0)	15 (11.7)	33 (9.2)	
Cohabiting with a partner, *n* (%)				
Yes	379 (73.0)	93 (77.5)	240 (70.0)	0.114
No	140 (27.0)	27 (22.5)	103 (30.0)	
Smoker, *n* (%)				
Yes	2 (0.4)	0 (0)	2 (0.6)	>0.999
No	542 (99.6)	128 (100)	355 (99.4)	
Highest level of education, *n* (%)				
No education	13 (2.5)	0 (0)	12 (3.4)	<0.001 ^a^
1st to 4th grade	207 (39.1)	32 (25.8)	152 (43.6)	
5th to 6th grade	114 (21.5)	22 (17.7)	79 (22.6)	
7th to 9th grade	126 (23.8)	34 (27.4)	78 (22.3)	
10th to 12th grade	64 (12.1)	33 (26.6)	25 (7.2)	
Higher education	6 (1.1)	3 (2.4)	3 (0.9)	
Occupational status in the last 12 months, *n* (%)				
Student	13 (2.4)	7 (5.5)	6 (1.7)	0.007
Housewife/househusband	187 (34.4)	33 (25.8)	122 (34.2)	
Employed/self-employed	265 (48.8)	74 (57.8)	166 (46.5)	
Unemployed or retired	78 (14.4)	14 (10.9)	63 (17.6)	
Household members, Mdn (P25–P75)				
Number of members living together (*n* = 544)	6.00 (5.00–7.00)	5.00 (5.00–7.00)	5.00 (5.00–7.00)	0.484
Number of members aged <5 years old (*n* = 542)	1.00 (0.00–1.00)	1.00 (0.00–1.00)	1.00 (0.00–1.00)	0.875
Number of members aged between 5 and 18 years old (*n* = 544)	3.00 (2.00–3.00)	3.00 (2.00–3.75)	3.00 (2.00–3.00)	0.894
Number members aged >18 years old (*n* = 544)	2.00 (2.00–3.00)	2.00 (2.00–2.00)	2.00 (2.00–3.00)	0.292
Household income in STN, *n* (%)				
<500	33 (7.7)	2 (2.0)	28 (10.3)	0.004
500–1000	43 (10.1)	7 (7.1)	16 (5.9)	
1000–2000	113 (26.5)	21 (21.2)	75 (27.5)	
2000–3000	135 (31.6)	31 (31.3)	95 (34.8)	
>3000	103 (24.1)	38 (38.4)	59 (21.6)	
Daily amount of food expenses, in STN, *n* (%)				
<50	22 (4.3)	2 (1.7)	17 (5.0)	<0.001
50–100	137 (26.7)	16 (13.6)	98 (29.0)	
100–200	284 (55.3)	77 (65.3)	185 (54.7)	
200–300	57 (11.1)	15 (12.7)	34 (10.1)	
>300	14 (2.7)	8 (6.8)	4 (1.2)	
Person responsible for preparing/cooking meals at home, *n* (%)				
Mother or father	463 (85.3)	103 (81.1)	307 (86.0)	0.188
Grandparent	34 (6.3)	8 (6.3)	24 (6.7)	
Children or others	46 (8.5)	16 (12.6)	26 (7.3)	
Home garden, *n* (%)				
Yes	73 (13.4)	27 (21.3)	41 (11.5)	0.006
No	471 (86.6)	100 (78.7)	317 (88.5)	

Note: The total in the first column corresponds to the characterization of all the enrolled children and adolescents’ families, which differs from the total for food insecurity status variable. This difference is justified by the presence of missing data. FI: food insecurity; Mdn: median; P25-P75: 25th and 75th percentiles; STN: São Tomé dobras (official national currency). ^a^: *p* value obtained after recoding the variable “highest level of education” into five categories: no education; 1st to 4th grade; 5th to 6th grade; 7th to 9th grade; and 10th to 12th grade or higher education.

**Table 2:** Children’s/adolescents’ characteristics according to food insecurity status.

	Total	Non-Severe FI	Severe FI	*p*-Value
Sex, *n* (%)				
Male	283 (51.8)	70 (54.7)	182 (50.7)	0.438
Female	263 (48.2)	58 (45.3)	177 (49.3)	
Age in years, Mean ± SD	10.82 ± 1.17	10.73 ± 1.22	10.81 ± 1.12	0.466
School grade, *n* (%)				
5th grade	307 (56.2)	73 (57.0)	216 (60.2)	0.535
6th grade	239 (43.8)	55 (43.0)	143 (39.8)	
Number of siblings (*n* = 542), Mdn (P25–P75)	3.00 (2.00−4.00)	3.00 (2.00–4.00)	3.00 (2.00–4.00)	0.546
Birth order, *n* (%)				
First-born	142 (29.7)	37 (32.5)	90 (29.4)	0.184
Second-born	115 (24.1)	27 (23.7)	75 (24.5)	
Third-born	93 (19.5)	22 (19.3)	57 (18.6)	
Fourth-born	60 (12.6)	16 (14.0)	34 (11.1)	
Fifth-born	35 (7.3)	10 (8.8)	22 (7.2)	
Sixth-born or other	33 (6.9)	2 (1.8)	28 (9.2)	
Maternal age at birth in years *(n* = 496), Mdn (P25–P75)	25.00 (21.00−31.00)	24.00 (20.00–29.00)	25.00 (21.00–32.00)	0.176
Birth weight in grams (*n* = 433), Mean ± SD	3219.32 ± 619.10	3308.06 ± 693.94	3171.60 ± 569.88	0.049
Very low birth weight, n (%)	2 (0.5)	0 (0)	1(0.3)	0.274
Low birth weight, n (%)	37 (8.4)	7 (6.8)	28 (9.6)	
Normal for gestational age, *n* (%)	371 (83.7)	85 (82.5)	245 (83.6)	
Macrosomic, n (%)	33 (7.4)	11 (10.7)	19 (6.5)	
Gestational age in weeks (*n* = 477)	39.50 ± 1.69	39.48 ± 1.24	39.50 ± 1.69	0.925
Preterm, *n* (%)	41 (8.5)	11 (10.6)	30 (9.4)	0.890
Term, *n* (%)	419 (87.3)	88 (84.6)	275 (86.5)	
Postterm, *n* (%)	20 (4.2)	5 (4.8)	13 (4.1)	
Breastfeeding, *n* (%)				
Yes	530 (98.5)	122 (98.4)	349 (98.3)	>0.999
No	8 (1.5)	2 (1.6)	6 (1.7)	
Breastfeeding duration in months (*n* = 487), Mdn (P25–P75)	18.00 (15.00–20.00)	18.00 (15.00–20.00)	18.00 (15.00–21.00)	0.162
Height-for-age z-score, at the interview (*n* = 539), Mean ± SD	−0.50 ± 1.08	−0.55 ± 1.19	−0.47 ± 1.04	0.524
Severely stunted, *n* (%)	6 (1.1)	4 (3.1)	2 (0.6)	>0.999 ^a^
Stunted, *n* (%)	33 (6.0)	7 (5.5)	21 (5.9)	
Normal height for age, *n* (%)	500 (91.6)	117 (91.4)	330 (93.5)	
BMI-for-age z-score, at the interview (*n* = 539), Mean ± SD	−0.66 ± 0.98	−0.59 ± 0.97	−0.70 ± 0.98	0.286
Severe thinness, *n* (%)	5 (0.9)	1 (0.8)	4 (1.1)	0.661 ^b^
Moderate thinness, *n* (%)	32 (5.9)	7 (5.5)	21 (5.9)	
Mild *thinness, n* (%)	147 (27.3)	35 (27.3)	95 (26.9)	
Normal weight, *n* (%)	331 (61.4)	77 (60.2)	220 (62.3)	
Overweight, *n* (%)	19 (3.5)	7 (5.5)	10 (2.8)	
Obesity, *n* (%)	5 (0.9)	1 (0.8)	3 (0.8)	
Dietary oral supplement in the previous 12 months, *n* (%)				
Yes	242 (55.1)	57 (60.0)	146 (49.2)	0.066
No	197 (44.9)	38 (40.0)	151 (50.8)	
Main source of child/adolescent nutrition, *n* (%)				
Own production, farming, hunting, or fishing	35 (6.4)	9 (7.0)	24 (6.7)	0.063
Purchases	494 (90.5)	119 (93.0)	320 (89.1)	
Exchanges (including exchanges for work), donations from friends/family or food assistance	17 (3.1)	0 (0)	15 (4.2)	
Eating breakfast before going to school? *n* (%)				
Yes	498 (91.7)	115 (90.6)	328 (91.9)	0.883
No	22 (4.1)	5 (3.9)	13 (3.6)	
Sometimes	23 (4.2)	7 (5.5)	16 (4.5)	
Eating a hot meal for lunch at school, *n* (%)				
Yes	510 (93.4)	118 (92.2)	337 (93.9)	0.509
No	36 (6.6)	10 (7.8)	22 (6.1)	
Frequency of hot school meals consumption? *n* (%)				
One time/week	26 (5.2)	3 (2.6)	21 (6.3)	0.145
Two times/week	33 (6.5)	10 (8.6)	19 (5.7)	
Three times/week	71 (14.1)	14 (12.1)	50 (14.9)	
Four times/week	60 (11.9)	19 (16.4)	34 (10.1)	
Daily	314 (62.3)	70 (60.3)	212 (63.1)	
Bringing snacks from home to school? *n* (%)				
Yes	208 (38.4)	52 (40.9)	134 (37.5)	0.789
No	269 (49.6)	61 (48.0)	180 (50.4)	
Sometimes	65 (12)	14 (11.0)	43 (12.0)	
Buying foods at school and/or near it? *n* (%)				
Yes	478 (87.7)	114 (89.1)	312 (87.2)	0.573
No	67 (12.3)	14 (10.9)	46 (12.8)	
Place of purchases, *n* (%)				
Inside the school	170 (37.9)	43 (40.6)	109 (37.1)	0.126
Outside the school (up to 100 m)	245 (54.6)	51 (48.1)	167 (56.8)	
Both or other places	34 (7.6)	12 (11.3)	18 (6.1)	
Frequency of those food purchases? *n* (%)				
Less than one time/week	82 (15.9)	22 (18.2)	52 (15.3)	0.516
One to two times/week	184 (35.6)	39 (32.2)	120 (35.3)	
Three to four times/week	153 (29.6)	40 (33.1)	99 (29.1)	
Daily	84 (16.2)	19 (15.7)	58 (17.1)	
More than once a day	14 (2.7)	1 (0.8)	11 (3.2)	
Money spent on those purchases in STN (*n* = 476), Mdn (P25-P75)	5.00 (2.00–6.00)	5.00 (2.00–7.00)	5.00 (2.00–6.00)	0.693
Drinking water while at school, *n* (%)				
Yes	490 (89.7)	122 (95.3)	317 (88.3)	0.022
No	56 (10.3)	6 (4.7)	42 (11.7)	
Source of that water, *n* (%)				
Tap	109 (34.8)	32 (39.0)	67 (33.2)	0.680
Bottled	15 (4.8)	4 (4.9)	10 (5.0)	
Fountain or well	141 (45.0)	32 (39.0)	97 (48.0)	
River or stream	38 (12.1)	10 (12.2)	22 (10.9)	
Other (rain, tank…)	10 (3.2)	4 (4.9)	6 (3.0)	
Treated water, *n* (%)				
Yes	120 (54.4)	27 (46.6)	81 (46.3)	0.972
No	143 (45.6)	31 (53.4)	94 (53.7)	
Mode of commuting in a typical week (Monday to Friday)				
From home to school, *n* (%)				
By foot	527 (96.7)	124 (96.9)	353 (98.3)	0.273
Transport (van, school bus, or motorcycle)	18 (3.3)	4 (5.1)	6 (1.7)	
Duration in minutes (*n* = 546)	20.00 (10.00–40.00)	20.00 (10.00–30.00)	20.00 (10.00–40.00)	0.437
From school to home, *n* (%)				
By foot	536 (98.2)	124 (96.9)	353 (98.3)	0.299
Transport (van, school bus, or motorcycle)	10 (1.8)	4 (3.1)	6 (1.7)	
Duration in minutes (*n* = 546), Mdn (P25–P75)	20.00 (10.00–40.00)	20.00 (10.00–35.00)	20.00 (10.00–40.00)	0.476
Daily time of physical activity and/or sport and/or active play in minutes (*n* = 546), Mdn (P25–P75)	120.00 (60.00–180.00)	90.00 (60.00–120.00)	120.00 (60.00–180.00)	0.421
Daily sleep duration in hours and minutes (*n* = 546), Mdn (P25–P75)	11:00 (10:00–11:00)	11:00 (10:00–11:00)	11:00 (10:00–11:00)	0.788

Note: The total in the first column corresponds to the characterization of the enrolled children and adolescents, which differs from the total (not shown) for food insecurity status variable. This difference is justified by the presence of missing data. BMI: body mass index; FI: food insecurity; Mdn: median; P25–P75: 25th and 75th percentiles; STN: São Tomé dobras (official national currency). ^a^: *p* value obtained after recoding the variable height-for-age into two categories: stunted (including severely stunted) and normal height-for-age. ^b^: *p* value obtained after recoding the variable height-for-age into four categories: severe or moderate thinness; mild thinness; normal weight; overweight or obesity.

**Table 3:** Nutritional intake of children and adolescents according to the level of food insecurity (*n* = 546).

	Total Mdn (P25–75) *	Non-Severe FI Mdn (P25–75) *	Severe FI Mdn (P25–75) *	*p*-Value
Total energy (kcal/day), Mean ± SD	1484.99 ± 574.84	1519.98 ± 626.99	1491.93 ± 576.83	0.645
Total fat (g/day)	45.07 (31.34–62.32)	47.55 (32.53–65.39)	44.69 (31.09–62.01)	0.437
Total fat (%TEV)	28.73 (23.42–35.53)	29.36 (23.20–36.66)	28.57 (23.42–35.37)	0.427
Saturated fat (g/day)	8.21 (5.21–11.94)	8.54 (5.94–12.17)	8.26 (5.95–12.03)	0.506
Saturated fat (%TEV)	5.32 (4.00–6.97)	5.43 (4.21–7.19)	5.32 (4.01–7.03)	0.609
Monounsaturated fat (g/day)	10.02 (6.16–14.77)	10.30 (5.96–14.67)	10.09 (6016–14.87)	0.940
Monounsaturated fat (%TEV)	6.35 (4.45–8.62)	6.11 (4.48–8.67)	6.52 (4.48–8.55)	0.736
Polyunsaturated fat (g/day)	13.68 (7.80–22.49)	13.77 (8.22–23.73)	14.08 (7.33–22.92)	0.650
Polyunsaturated fat (%TEV)	8.78 (4.45–8.62)	8.88 (5.88–12.50)	8.65 (5.88–12.86)	0.800
Carbohydrates (g/day), Mean ± SD	202.87 ± 86.39	209.89 ± 92.54	207.85 ± 84.11	0.819
Carbohydrates (%TEV), Mean ± SD	55.97 ± 9.90	55.64 ± 9.69	56.16 ± 9.88	0.603
Total sugars (g/day)	37.67 (13.85–68.51)	37.96 (15.89–68.20)	38.00 (13.26–70.47)	0.676
Total sugars (%TEV)	10.62 (4.28–19.15)	10.00 (5.05–19.86)	11.02 (3.91–19.39)	0.697
Total fiber (g/day)	12.15 (7.46–18.01)	12.02 (7.35–18.18)	12.34 (7.70–18.19)	0.671
Soluble fiber (g/day)	0.02 (0.00–24.38)	0.02 (0.00–19.89)	0.02 (0.00–24.38)	0.568
Protein (g/day), Mean ± SD	44.17 ± 17.73	45.01 ± 18.73	44.34 ± 17.28	0.713
Protein (g/kg/day), Mean ± SD	1.36 ± 0.58	1.41 ± 0.62	1.37 ± 0.58	0.590
Protein (%TEV), Mean ± SD	12.14 ± 2.95	12.13 ± 2.99	12.19 ± 2.95	0.845
Cholesterol (mg/day)	48.14 (28.18–83.95)	48.65 (24.79–83.14)	51.84 (30.40–86.27)	0.478
Vitamin A (µg RE/day)	118.21 (53.27–199.69)	115.36 (52.54–202.32)	121.98 (54.37–199.85)	0.860
Retinol (µg/day)	99.56 (50.72–213.93)	98.28 (50.50–264.19)	102.03 (51.78–193.20)	0.364
Beta-carotene (µg/day)	1405.86 (620.80–2543.39)	1429.06 (637.21–2600.15)	1378.22 (651.13–2486.35)	0.606
Vitamin B-1 (mg/day)	0.49 (0.34–0.69)	0.49 (0.36–0.71)	0.49 (035–0.70)	0.704
Vitamin B-2 (mg/day)	0.42 (0.25–0.63)	0.42 (0.23–0.67)	0.43 (0.26–0.63)	0.877
Vitamin PP (mg NE/day)	9.39 (6.12–13.37)	9.29 (5.54–13.32)	9.46 (6.18–13.52)	0.449
Vitamin B-6 (mg/day)	0.74 (0.52–1.04)	0.74 (0.47–1.04)	0.76 (0.55–1.06)	0.539
Vitamin B-12 (µg/day)	3.12 (1.32–5.93)	3.00 (1.03–5.65)	3.27 (1.32–6.05)	0.345
Vitamin C (mg/day)	49.17 (27.67–79.33)	48.97 (25.29–77.55)	48.89 (27.93–79.52)	0.871
Vitamin D (µg/day)	6.77 (2.70–11.70)	6.51 (2.40–11.33)	6.77 (2.79–12.32)	0.344
Vitamin E α-tocopherol (mg/day)	7.05 (3.56–12.59)	8.12 (3.69–13.21)	7.00 (3.74–12.73)	0.547
Folate DFE (µg/day)	882.18 (58.41–121.58)	81.11 (60.66–124.46)	86.06 (58.47–170.49)	0.882
Vitamin K (µg/day)	2.81 (0.54–5.52)	2.66 (0.46–5.33)	2.84 (0.63–6.02)	0.262
Iron (mg/day)	4.13 (3.12–5.32)	4.16 (2.97–5.68)	4.18 (3.16–5.32)	0.606
Magnesium (mg/day)	141.71 (107.90–188.43)	134.48 (104.62–190.73)	145.14 (110.51–188.84)	0.413
Calcium (mg/day)	161.59 (100.94–289.50)	195.99 (112.44–329.60)	158.25 (100.54–274.75)	0.067
Zinc (mg/day)	3.22 (2.29–4.39)	3.24 (2.29–4.66)	3.28 (2.33–4.49)	0.966
Copper (mg/day)	0.14 (0.04–0.32)	0.11 (0.04–0.28)	0.16 (0.04–0.34)	0.113
Phosphorus (mg/day)	455.32 (317.31–599.18)	463.46 (319.58–629.34)	460.15 (318.27–604.00)	0.932
Potassium (mg/day)	1321.16 (862.01–1892.49)	1303.67 (882.59–1957.44)	1319.51 (903.47–1877.45)	0.797
Selenium (µg/day)	20.32 (7.63–40.28)	16.65 (2.42–40.45)	20.32 (7.67–40.64)	0.438
Sodium (mg/day)	1141.92 (779.16–1700.48)	1205.76 (809.63–1817.06)	1139.34 (786.06–1663.33)	0.373

Note: The total in the first column corresponds to nutritional intake of all children and adolescents, which differs from the total (not shown) for food insecurity status variable. This difference is justified by the presence of missing data. DFE: dietary folate equivalents, FI: food insecurity; M: mean; Mdn: median; NE: niacin equivalents; P25–P75: 25th and 75th percentiles; RE: retinol equivalents; SD: standard deviation; TEV: total energy value; *: For continuous variables with a normal distribution, the mean (M) and standard deviation (SD) are presented.

**Table 4:** Adjusted prevalence of nutritional inadequacy among children and adolescents.

	Age Group (Years)	Adjusted Prevalence of Nutritional Inadequacy (%)
Protein		
*n* = 251	9–10	0.0
*n* = 288	11–15	1.2
Vitamin A		
*n* = 251	9–10	97.4
*n* = 288	11–15	100.0
Vitamin B-1		
*n* = 251	9–10	35.6
*n* = 288	11–15	73.9
Vitamin B-2		
*n* = 251	9–10	96.8
*n* = 288	11–15	99.7
Vitamin PP		
*n* = 251	9–10	3.7
*n* = 288	11–15	37.4
Vitamin B-6		
*n* = 251	9–10	69.6
*n* = 288	11–15	96.6
Vitamin B-12		
*n* = 251	9–10	0.6
*n* = 288	11–15	3.9
Vitamin C		
*n* = 251	9–10	16.5
*n* = 288	11–15	52.4
Vitamin D		
*n* = 247	9–10	70.0
*n* = 283	11–15	71.1
Vitamin E α-tocopherol		
*n* = 250	9–10	13.1
*n* = 285	11–15	60.4
Folate DFE		
*n* = 251	9–10	96.9
*n* = 288	11–15	98.4
Iron		
*n* = 251	9–10	99.8
*n* = 288	11–15	100.0
Magnesium		
*n* = 251	9–10	9.8
*n* = 288	11–15	86.2
Calcium		
*n* = 251	9–10	100.0
*n* = 288	11–15	100.0
Zinc		
*n* = 225	9–10	99.7
*n* = 284	11–15	100.0
Copper		
*n* = 244	9–10	100.0
*n* = 279	11–15	100.0
Phosphorus		
*n* = 94	9–10	23
*n* = 283	11–15	100.0
Selenium		
*n* = 135	9–10	29.1
*n* = 198	11–15	74.7

DFE: dietary folate equivalents; NE: niacin equivalents; RE: retinol equivalents.

**Table 5:** Summary of households’ food security situation according the HFIAS questionnaire.

	Total	Non-Severe FI	Severe FI	*p*-Value
HFIAS-related Domains, *n* (%)				
Anxiety and uncertainty (about food supply)				
Yes	416 (76.5)	62 (48.4)	302 (84.1)	<0.001
No	128 (23.5)	66 (51.6)	57 (15.9)	
Insufficient quality (including variety and food preferences)				
Yes	506 (93.0)	99 (77.3)	351 (97.8)	<0.001
No	38 (7.0)	29 (22.7)	8 (2.2)	
Insufficient food intake and inherent physical consequences				
Yes	491 (90.3)	79 (61.7)	358 (99.7)	<0.001
No	53 (9.7)	49 (38.3)	1 (0.3)	
HFIAS Score (*n* = 464), Mean ± SD	13.57 ± 6.67	6.40 ± 4.52	16.18 ± 5.27	<0.001
HFIA prevalence, *n* (%)				
Food secure	25 (5.1)			
Mildly food insecure	8 (1.6)			
Moderately insecure	95 (19.5)			
Severely food insecure	359 (73.7)			

FI: food insecurity; HFIA: household food insecurity access; HFIAS: Household Food Insecurity Access Scale; M: mean; SD: standard Deviation.

**Table 6:** Multivariable binary logistic regression for sociodemographic, lifestyle, and nutritional predictors of severe food insecurity of participants´ households.

	Variables	OR	95% CI	*p*-Value
Household	Head of the household´s sex			
	Male	Reference		
	Female	5.505	2.209–13.721	<0.001
	Head of the household’s age in years	1.045	1.008–1.084	0.017
	Household income in STN			
	<1000	Reference		
	1000–3000	0.786	0.316–1.956	0.605
	>3000	0.380	0.141–1.026	0.056
	Home garden			
	No	Reference		
	Yes	0.343	0.157–0.750	0.007
Child/adolescent	Sex			
	Male	Reference		
	Female	1.823	0.980–3.389	0.058
	BMI-for-age z-score	0.768	0.560–1.053	0.101
	Drinking water while at school			
	No	Reference		
	Yes	0.311	0.077–1.252	0.100
Nutritional Intake	Carbohydrates (g/day)	0.995	0.991–1.000	0.039
	Vitamin B-1 (mg/day)	1.702	0.831–3.484	0.146
	Iron (mg/day)	0.735	0.580–0.931	0.001
	Magnesium (mg/day)	1.013	1.004–1.021	0.003

Note: for this logistic regression model, the following variables were initially considered: family relationship of the student´s guardian, age, sex, level of education and occupational status of the head of the household, number of household members living together, household income (recoded), average daily amount of income spent on food, owning a home garden, children’s/adolescent´s sex, age, birth weight, and current BMI-for-age, drinking water while at school, total energy, total fat, protein, carbohydrates, vitamins A, B-1, B-2, PP, B-6, B12, C, D, E, and K, folate, iron, magnesium, calcium, zinc, copper, phosphorus, potassium, selenium, sodium, and being a misreporter. Area under the curve = 0.749. BMI: body mass index; CI: confidence intervals; OR: odds ratio; STN: São Tomé dobras (official national currency).

## Data Availability

Data are available upon request. The data are not publicly available due to confidentiality and privacy considerations.

## Appendix A. Crude Prevalence of Nutritional Inadequacy among Children and Adolescents

**Table A1:** Crude prevalence of nutritional inadequacy among children and adolescents.

	Age Group (Years)	Crude Prevalence of Nutritional Inadequacy (%)
Protein		
*n* = 251	9–10	5.2
*n* = 288	11–15	14.2
Vitamin A		
*n* = 251	9–10	87.6
*n* = 288	11–15	99.7
Vitamin B-1		
*n* = 251	9–10	51.8
*n* = 288	11–15	77.4
Vitamin B-2		
*n* = 251	9–10	88.4
*n* = 288	11–15	96.9
Vitamin PP		
*n* = 251	9–10	25.5
*n* = 288	11–15	47.9
Vitamin B-6		
*n* = 251	9–10	62.2
*n* = 288	11–15	87.5
Vitamin B-12		
*n* = 251	9–10	21.5
*n* = 288	11–15	22.2
Vitamin C		
*n* = 251	9–10	37.8
*n* = 288	11–15	59.0
Vitamin D		
*n* = 247	9–10	66.4
*n* = 283	11–15	68.2
Vitamin E α-tocopherol		
*n* = 250	9–10	37.2
*n* = 285	11–15	63.5
Folate DFE		
*n* = 251	9–10	85.3
*n* = 288	11–15	96.2
Iron		
*n* = 251	9–10	93.2
*n* = 288	11–15	99.3
Magnesium		
*n* = 251	9–10	23.5
*n* = 288	11–15	78.1
Calcium		
*n* = 251	9–10	98.4
*n* = 288	11–15	99.7
Zinc		
*n* = 225	9–10	100.0
*n* = 284	11–15	100.0
Copper		
*n* = 244	9–10	100.0
*n* = 279	11–15	100.0
Phosphorus		
*n* = 94	9–10	100.0
*n* = 283	11–15	100.0
Selenium		
*n* = 135	9–10	100.0
*n* = 198	11–15	100.0

DFE: dietary folate equivalents; NE: niacin equivalents; RE: retinol equivalents.
